# Baby-box schemes in England: parent and practitioner experiences, and recommendations

**DOI:** 10.1186/s12887-020-02064-2

**Published:** 2020-04-11

**Authors:** Helen L. Ball, Catherine E. Taylor

**Affiliations:** Infancy & Sleep Centre, Department of Anthropology, Durham University, Durham, DH1 3LE UK

**Keywords:** Infant health, Baby boxes, SIDS and SUDI, Health service partnerships, parent education

## Abstract

**Background:**

Programmes offering carboard baby boxes to parents in England began in some NHS Trusts in 2016. This study aimed to examine the strengths and weaknesses of English baby-box schemes as experienced by healthcare providers and parents.

**Methods:**

An independent mixed-methods evaluation was conducted via telephone interviews and online surveys with healthcare providers and parents in all 7/9 NHS regions of England where baby-box schemes were established 2016–2019. Participants responded to requests circulated electronically by NHS Research & Design Departments, and infant health organisations in England. The objectives were to identify how parents and healthcare providers understood and experienced baby-box schemes implemented in England to date, and to produce recommendations for organisations considering involvement in future schemes.

**Results:**

Baby-box schemes changed over time, and were complex to run and monitor. Both parents and practitioners were misinformed about their purpose and origins. Partnerships with a commercial box-provider reduced the investment needed to run a baby-box scheme, and offered potential benefits to staff regarding engagement with families via online education and face-to-face contact around handover of boxes, but carried unforeseen costs. Of particular concern was the box-provider’s access to parent personal details being promoted by NHS staff and parents’ lack of awareness; the hidden costs incurred by NHS facilities of running a box-scheme; and the costs incurred by parents in accessing their ‘free’ box. Sixteen recommendations are proposed for healthcare providers and organisations considering commercial - health-provider baby-box partnerships in future.

**Conclusions:**

Many assumptions exist about the origins and purpose of baby-boxes; this misinformation needs correcting, especially as it relates to infant death reduction and safe infant sleep. Baby-box schemes take multiple forms from those motivated by social welfare to those motivated by commercial profit. The English experience of partnership schemes between healthcare facilities and commercial box-providers reveals some success stories, along with multiple points of ambiguity, unanticipated difficulty, and concerns for infant safety.

## Background

A flurry of baby-box schemes appeared in English NHS Trusts from late 2016, generating debate about their strengths and weaknesses [1–3]. Between 2016 and 2019 these schemes proliferated, and underwent substantial changes, while a very different type of scheme was implemented in Scotland [4]. This report of an independent evaluation of baby-box schemes in England (conducted 2018–19) discusses the experiences of practitioners who instigated and executed these schemes, and of parent recipients, and offers recommendations for healthcare providers considering implementing future baby-box schemes.

### History of baby-boxes

The trend to provide parents with cardboard baby-boxes containing infant care supplies began in one London hospital in mid-2016, [5] and soon spread across England. Around the same time the Scottish Government launched a cardboard baby-box scheme (January 2017) that began with a pilot programme in two locations, [6] and expanded to the whole of Scotland in 2018 [7]. In addition to offering new parents a box of infant care items, a key feature of the baby-boxes in both England and Scotland was the inclusion of a fitted mattress inside the cardboard box and the promotion of baby-boxes as safe infant sleep spaces, with claims they would reduce the risk of sudden infant death syndrome (SIDS) [8–10].

Boxes of free infant care items have long been offered to pregnant women in Finland, [11] and Finnish public health officials confirm they began to incentivise prenatal care, [12] not to reduce infant deaths [13]. However baby-boxes were erroneously linked to SIDS-reduction in widely-cited UK media reports [14]. Although Finland’s SIDS/SUDI rates are low, rates in Denmark and Sweden are equally low without any baby-boxes, but with similar universal health care systems, social safety nets, and paid maternity leave policies [15]. Dramatic SIDS-reduction in England & Wales between 1988 and 2017 resulted from evidence-based research, with international case-control studies forming the basis for infant safe sleep recommendations, also without cardboard baby-boxes [15].

Despite growing popularity of baby-box schemes world-wide, [16] little research has explored parental use of cardboard baby-boxes, or how baby-box schemes have been implemented. Three US studies have been reported to date exploring usage prevalence, [17] usage intent, [18] and actual use only in the first 72 h [19]. A preliminary evaluation of the Scottish baby-box scheme offered initial insights into the “potential impacts of the scheme and possible barriers to achieving those impacts”, [6] however no firm conclusions were drawn, and there have been no published evaluations of baby-box schemes implemented in England.

In contrast to the cardboard baby-box schemes described here, low-sided ventilated plastic baby boxes known as Pepi-Pods are being implemented in New Zealand and Australia, particularly among indigenous communities with high infant mortality rates, alongside a Maori woven-flax variant known as a Wahakura [20–24]. Sometimes called ‘safe sleep enablers’ these devices can be used in the parents’ bed to ameliorate hazardous bed-sharing [24]. The Pepi-Pod and Wahakura programmes are intentionally designed to improve infant sleep safety and reduce infant mortality and therefore have very different origins and implementation strategies than the cardboard baby-box schemes addressed in this paper.

### Types of baby-box schemes

**Fig. 1:**
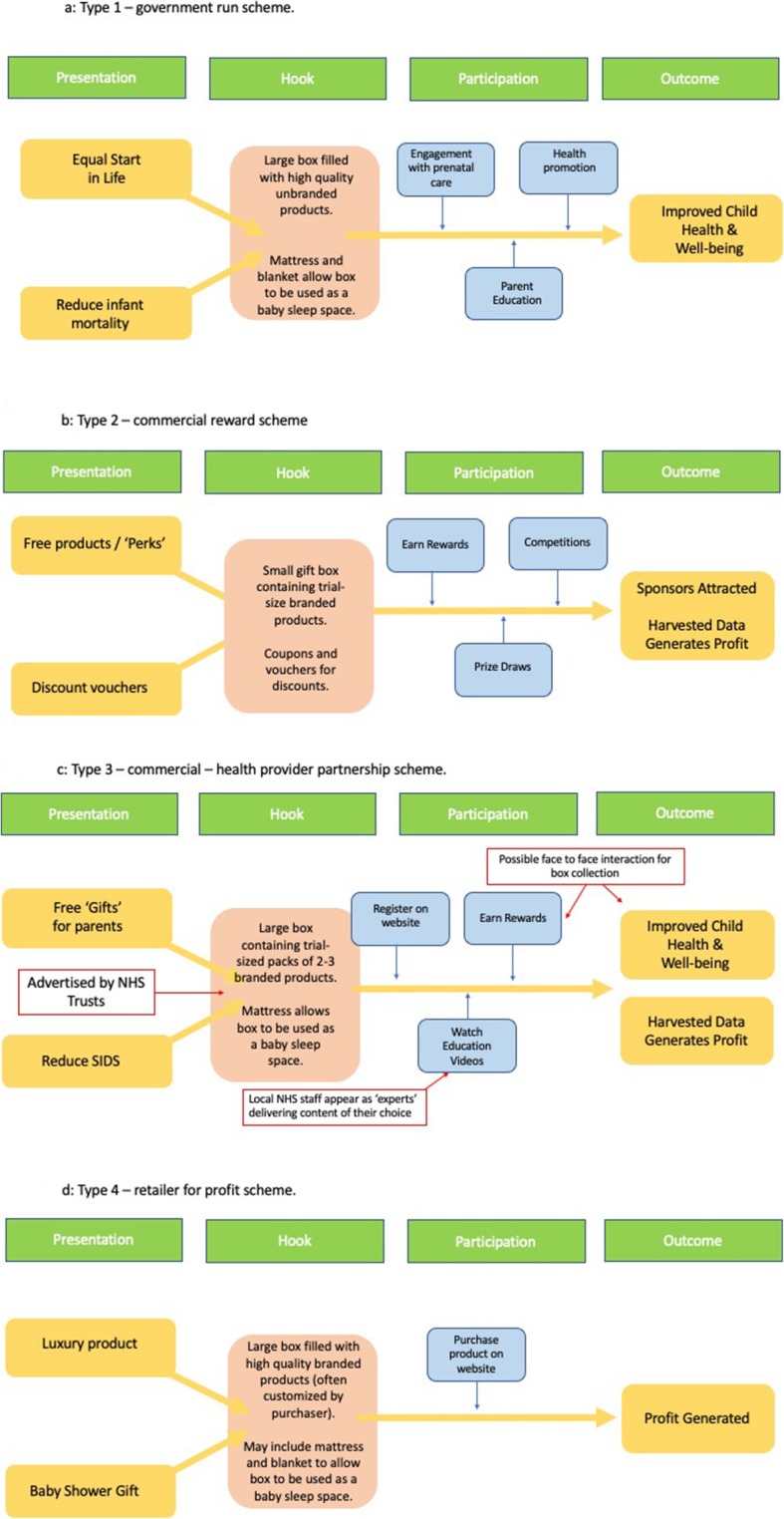
(**a**-**d**). Types of Baby Box schemes

There are 4 types of cardboard baby-box schemes (Type-1-4) currently operating, ranging from government-funded initiatives to direct commercial sales. The key features of these schemes are summarized in Fig. 1a-d. ‘Type-1’ government-funded schemes generally invoke ‘Start of Life Equity’ and ‘Reduced Infant Mortality’ values. High-quality unbranded infant clothing and essential products are distributed in a custom designed box with a fitted mattress that can be used as a portable infant sleep space. Boxes serve as rewards for engagement with early prenatal care, and provide opportunities for parental education via face-to-face conversation, or leaflets enclosed within the box. The intended outcome of Type-1 programmes is improved child health and well-being.

In ‘Type-2’ schemes free cardboard baby-boxes incentivise expectant parents to sign-up for baby-clubs or ‘reward’ websites. Reward-based commercial box schemes typically distribute small quantities of branded products (e.g. ‘trial-packs’) and discount coupons. The ‘baby-box’ may be a packaging or gift box, and may not include a mattress. Recipients are rewarded for supplying contact and other details that can be used for promotion of products and services. The intended outcome of Type-2 schemes is profit via the harvesting of data which is sold to sponsors and other third parties.

‘Type-3’ schemes are a hybrid of Type-1 and Type-2. In these schemes baby-boxes are promoted via healthcare facilities as a ‘safe-sleep space’, a gift, or a reward. To obtain it parents must sign-up to a commercial ‘rewards’ website. Box-contents involve trial-size products and discount coupons. The full-size box has a fitted mattress and a sheet. Parents may have the option to collect their box for free from a healthcare facility, or pay the box-provider a fee for delivery. The box is a reward for supplying contact data, but its collection is also an opportunity to bring parents into healthcare hubs. The intended outcome is to generate profit for the commercial partner, and face-to-face engagement for health-providers to improve child health and well-being.

‘Type-4’ schemes involve the direct sale of luxury products, often marketed as gifts for expectant parents via high-street and online retailers. Numerous companies offer baby-box products, with the majority including a mattress and varying amounts of infant care items/clothing according to price. Although Type-4 boxes are widely available, they are expensive and beyond the financial reach of many parents, although some employers offer them as gifts to staff [25].

### Overview of baby-box schemes in England

Most baby-box schemes in England involve Type-3 partnerships between healthcare facilities and a commercial box-provider. The first such partnership was established in 2016 at a London hospital. English baby-box schemes were initially free for parents, and offered access to an online education platform and a baby-box containing a mattress, sheet, and some infant-care products. To obtain a box parents registered on the US-based box-provider’s website and consented to their data being captured. They then gained access to free educational videos for their region (based on postcode), and completed a multiple-choice quiz. Access to this education platform was only provided to parents who completed the registration process to receive a box. Successful quiz completion led to a certificate for a free baby-box to be obtained from the participating hospital or health centre [8, 9].

The education platform (website) hosted video content by medical and non-medical practitioners in multiple countries, covering pregnancy, safe sleep, breastfeeding, maternal mental health, and child development. Early healthcare partners scripted and narrated educational videos to suit local priorities. Videos were uploaded by the box-provider to the parent-education website. If local videos on safe infant sleep, or how to use the baby-box were absent, US-made videos were sometimes substituted, offering safe sleep recommendations that differed from guidance in England (although UK specific guidance is now available). As video content accrued the option to script and film local videos ceased and healthcare partners selected educational videos from the existing catalogue. Healthcare providers promoted the scheme to expectant parents, displaying posters, engaging with the media, and informing parents of the scheme.

During 2018 changes were made by the box-provider to the box contents and educational website. By 2019 the educational platform was extensively altered and re-branded. Parents were now encouraged to complete courses at different stages of their child’s life to unlock ‘rewards’ in the form of discounts for products and services. Baby-boxes still featured in the first ‘reward’ but were no longer free; shipping fees were required to obtain a box, and the products were now sponsored by a supermarket chain.

### English baby-box scheme evaluation

We undertook an independent evaluation to understand the English experience of Type-3 baby-box schemes, and provide recommendations for healthcare providers considering such a scheme. In early 2018 when this project commenced 24 NHS facilities in England were confirmed participants in baby-box schemes in seven of the nine English NHS regions: 4 in Greater London, 3 in South East, 4 in West Midlands, 3 in North West, 2 in Yorkshire & Humber, 1 in East Midlands, and 4 in East of England. No schemes were running in North East or South West regions of England.

## Methods

Institutional ethical approval was received for this research to be conducted (see Declarations). Data on English baby-box schemes were obtained via telephone interviews with practitioners, anonymous online surveys with practitioners and parents, and via direct observation of online content during 2018–2019. Research offices in the 24 NHS Trusts where baby-box schemes operated were notified of the evaluation and asked to circulate details to staff inviting them to participate in interviews; 8/24 confirmed by email that they had done so. Participation was voluntary, no incentives were offered, and participants confirmed informed consent. Interviews explored how organisations became involved in a baby-box programme, how programmes had been tailored for service users in each area, what the successes and challenges the programme had been, what feedback had been received from service users, whether the programme affected the delivery of services, and the interviewee’s opinions and experiences of programme.

Online surveys conducted in Spring 2019 supplemented the interview data with views and experiences from a wider range of healthcare providers and parents. The surveys were hosted on the JISC Online Surveys website, a secure higher education platform approved by research institutions. Invitations to participate were circulated via email and social media to contacts of the Durham Infancy & Sleep Centre, the Lullaby Trust, and Unicef UK Baby Friendly Initiative as organisations that provide information about infant care in the UK. Contacts confirmed they cascaded survey information to others in their networks. Survey completion was voluntary and again, no incentives were offered. Parents/carers were eligible to participate if they resided in England and were familiar with a local baby-box programme (whether they had received a box or not). Practitioners were eligible if they worked as a healthcare provider (including support workers, charity workers and volunteers) and were familiar with an existing or proposed baby-box programme in England.

Employees of organisations creating or selling baby-boxes were excluded from both surveys; anyone submitting responses that failed eligibility criteria automatically left the survey. A core set of multiple-choice survey questions were compulsory (10 for parents, 18 for practitioners) while free text responses were optional. Adaptive questioning (where sub-questions are hidden or revealed (known as skip-logic) depending on the response to previous questions) was used to tailor the survey to the individual respondent and avoid redundant questions. Incomplete responses were not retained. Practitioner and parent survey questions can be found in the Supplementary Materials. Frequencies and percentages were calculated to describe the responses to closed categorical questions, and an inductive thematic approach was taken to coding and analysing both interview data and free text responses to survey questions using NVivo qualitative data analysis software.

## Results

Participant numbers are summarised in Table 1. Practitioner telephone interviews lasting 20–40 min, were completed between July 2018 and March 2019. Interviewees held a variety of positions, and were employed across 6 health regions with direct experience of 8 different baby-box schemes. Half of the interviewees had been involved in setting-up a baby-box programme, and the other half were involved in implementation.

**Table 1:** Summary table of participants

Participant numbers		
	Responses received	Eligible responses
Parent/carer survey	81	77
Practitioner survey	60	60
Interviews	10 registrations of interest^a^	8 completed interviews
Practitioner interviewee position (e.g. NHS Job Role)		
n	Position title	
2	Infant Feeding Lead	
1	Community Midwife	
1	Birth Centre Matron (Manager)	
1	Consultant Obstetrician and Gynaecologist	
1	Lifestyle and Wellbeing Service Manager	
1	Community Support Worker	
1	Injury Prevention Officer	
Practitioner survey respondent position description (e.g. NHS Job Role)		
n	Position title	
28	Community Health Practitioner	
14	Clinical Practitioner	
5	Local Authority Employee	
5	Peer Support, Volunteer or Charity Worker	
3	Community Support Worker	
1	Health Services Commissioner	
4	Other	

^a^two failed to respond to the interview invitation

Sixty responses were received to the practitioner survey; no responses were excluded. Respondents primarily described themselves as healthcare practitioners working in a community (47%) or clinical setting (23%) (Table 1). Responses were received from practitioners in all nine regions of England with a bias towards the North West of England (30%). Eighty-one responses were received to the parent survey; four responses were completed by health practitioners and excluded. All parent participants were female and 97% (75/77) described themselves as White. Responses were received from all nine regions of England with a bias towards the East of England (40%). Due to the distribution of the surveys by partner-organisations an accurate denominator could not be determined to allow calculation of a response rate.

### Practioner experiences and views of baby-box schemes

All practitioners interviewed, and 83% (50/60) of those surveyed, were directly involved in decision-making around or implementation and operation of a baby-box scheme, while 17% (10/60) had second-hand knowledge via colleagues or the media. 80% (48/60) of survey respondents had seen or handled a baby-box and its contents. Few practitioners identified the scheme with which they were familiar as being run by a commercial company (17%, 10/60); 42% (25/60) thought the boxes originated from an NHS or council organisation, while a quarter (25%, 15/60) did not know who the provider was. Three practitioners (5%) named the provider but believed they were a charity.

#### Initiating and implementing the scheme

Practitioner interviews confirmed that the online education platform offered by the box-provider, and the opportunity to interact with parents who collected a box in person, were the main attractions of the scheme: free ‘safer sleep box’, potential to promote other services, and no-cost opportunity to be involved were other motivations.

“It was the online platform and also looking at the box, it seemed to conform to all the relevant safety standards like the boxes in Scotland but it gave us the opportunity to get something online that wouldn’t normally be there at no cost to us.”

“Obviously it’s got that feel good factor of people liking it because people feel that you are giving them a gift but actually to be able to reach out postnatally and give them what they need (services), that was more attractive to us.”

“Our aim was to use the scheme as an engagement tool, to get people into the hubs and to promote what’s available at the hubs.”

However practitioner accounts of the purpose of the baby-box scheme differed by level of involvement. For those initiating a specific scheme the opportunity for education and engagement with services was paramount, while those who undertook implementation placed a stronger emphasis on the box as a tool to reduce SIDS/infant mortality.

“I think it was [introduced] for perinatal targets and outcomes, trying to reduce the UK stats for SIDS and looking at what other countries do around sleep safety so using boxes, like they do in Finland, and when you compare the UK rates to their rates, theirs are a lot lower than ours so I believe that’s where it came from.”

One publicised benefit of the Type-3 schemes as implemented in England was that participation was ‘free’ for healthcare facilities, however interviewees identified multiple costs. Staff-time invested in the set up and management of a scheme was substantial, and required (but was not limited to) attendance at decision-making and roll-out meetings; creating video scripts and filming; receiving and storing flat-packed boxes, and box assembly; handing out boxes to eligible parents; and face-to-face guidance about box-use. Space was needed for storing the flat-packed and assembled boxes. Practitioners reported that running a baby-box scheme burdened healthcare resources, and hospital box distribution diminished over time due to capacity issues.

“Our local midwife and health visitor teams are understaffed and overworked; our Trust is millions of pounds in the red; staff are overworked and demoralised. When will they have time to organise and store huge numbers (5000 births) of boxes and products?”

#### The baby-boxes

In interviews and survey responses practitioners emphasised concerns regarding the lack of safety standards for cardboard infant sleep spaces, and the quality and provenance of the materials used: one practitioner noted: “We had some issues relating as to whether relevant safety standards were adhered to, the boxes were modified as a result of this.” Community practitioners also reported on how parents used their baby-box; the most frequent observed uses were as a toy box/storage container (*n* = 13), an occasional daytime sleep space at home (*n* = 9) or in someone else’s home (*n* = 4), as a primary sleep space (*n* = 2), or as a dog bed (*n* = 1).

“Quite a few [are] disappointed when they actually get the box as [they] feel it is not very substantial as they expected - some have not used at all or used for storage of other items, some have left at a grandparent’s house for when they visit.”

“I worked with many parents who were homeless or staying in one room bedsits/with family. Many in abject poverty and unsafe bedsharing conditions because of the inability to change the room set up/bed or because of smoking/alcohol/drugs. … the area I worked in benefitted from the scheme and this is the reason I got involved, for those parents.”

“[Parents were] disappointed the box was a box! Initial images for the scheme showed the box to be full of freebies so some parents were disappointed.”

“[One of them said] Oh, is that it? Where’s the rest of it?”

There were mixed feelings about the infant-care products provided in the boxes; 46% (17/60) of practitioners surveyed found them unsatisfactory, while 54% (20/60) were [somewhat] satisfied. Comments indicated the products were sparse, poor quality, inappropriately branded, or items the Trust did not promote (e.g. new-born hats). Others felt the free products attracted parents to the scheme. Interview and survey responses indicated that box contents changed over time and differed between regions; in some locations hospitals supplemented the contents from the box-provider.

Although some practitioners (*n* = 5) felt in-person box collection should be compulsory and some (*n* = 5) described how parents benefitted from face-to-face interactions, others noted barriers to collecting boxes from health facilities such as difficulty carrying a large box on public transport, distance to travel for collection, and restricted timings for when boxes could be collected. One respondent noted: “[Parents] like the concept but the poor contents and onerous conditions re. obtaining the box put them off.”

Of practitioners surveyed 67% (40/60) responded that parents were actively encouraged to use the box as a baby sleep space, however 42% (12/29) of those who had received feedback from parents were concerned how boxes were used. Examples given included lids placed on boxes with babies inside (*n* = 2), boxes placed on unsuitable surfaces (*n* = 4), and modifications to boxes by parents before use: three respondents witnessed the mattress being replaced with memory foam, a sleep pod, or an adult pillow. Others reported the box being tilted (*n* = 1) and the addition of soft-toys (*n* = 2), pillows (*n* = 1) or extra blankets (*n* = 1). These observations caused practitioners to question the safety of distributing baby-boxes without face-to-face guidance.

#### The online education

Practitioners familiar with the box-provider’s educational platform (34/60) indicated three main topics were addressed: safe sleeping and SIDS (94%, 32/34); baby-box safety (62%, 21/34); and general infant care (50%, 17/34). Other topics reported included antenatal care, infant feeding, and maternal mental health. Practitioner interviewees explained that the educational information delivered online for their locale may have been decided by the midwifery team, the box-provider, or the hospital management. The information content was described by practitioners as originating from multiple sources, most frequently the box-provider (38%, 13/34), the local NHS trust (35%, 12/34), national NHS (24%, 8/34), or a national charity (18%, 6/34). Interviewees appreciated the option to develop their own video content: “We picked what we wanted, we did the scripts and then they came and filmed; it was completely our agenda.” But one practitioner expressed discomfort with the label of ‘expert’ assigned to those appearing in the videos, noting: “I’m certainly not happy that I’m on there as the expert because I am not, [ …] I do not want to be on there as the expert!”

Although a key feature of the scheme involved parents viewing educational videos before obtaining a certificate/code to claim their free baby-box, several practitioners were unhappy that parents could avoid this, feeling it undermined any benefit of the scheme. “It’s easy for parents to click past/skip and subsequently not receive the information.” On the other hand, the need to complete the online education was questioned by some survey participants (*n* = 8) who felt this excluded non-English speakers or those lacking internet access.

#### Other feedback on the schemes

Practitioner interviewees and survey respondents had other concerns about baby-box schemes in England. The most frequent of these was unease at healthcare providers encouraging health-service users to provide personal data online to a US-based commercial organisation who may use or sell the data for marketing purposes. Most survey respondents (77%, 46/60) were concerned about parents’ personal data being used by the company or their partners, and a large majority (82%, 49/60) felt the NHS should not facilitate access to patients by commercial entities [26]. Recent prosecutions of companies who had accessed service-user data via UK maternity services were clearly in minds of many [27, 28]. The need for rigorous data protection agreements with collaborating companies was suggested, although this may undermine the box-provider’s profit model and thus their willingness to participate.

Since the implementation of the first baby-box schemes in England in 2016, numerous changes were implemented by both the box-provider and other partners. Practitioners were unhappy with these unanticipated changes, feeling they undermined any potential value of the schemes with which they were involved.

“The boxes were initially collected from our Children’s Centres, which we welcomed as an engagement opportunity. Without consultation, the model has now changed, and they are posted direct at cost to parents.”

“I work in a really deprived area. We were excited about … using the boxes as an incentive for people to do the online course and learn more about keeping their baby healthy and happy. However, once we saw the contents and the stringent procedures put in place re obtaining the box … we struggled to encourage people to participate.”

“There was joint promotion between local NHS trust and Children’s Centres. The NHS has pulled out, but Children’s Centre are still promoting. As an NHS support worker I have to tell our families that we are no longer recommending them and that if they did choose to use the baby-box only do so while supervised in the day time. This is going against the promotions that Children’s Centres are doing, and staff are still viewing them as being amazing and safe.”

“These schemes are turning into a mechanism to push products and brands and harvest families’ personal data. The online “education” is very mixed and much of it is very poor. The box is widely promoted as a means to provide “safe sleep“ but the box actually separates the mother and baby and makes breastfeeding more difficult, which really DOES raise baby’s risk of cot death. Not to mention that a cardboard box is a complete fire hazard!”

### Parent experiences and views of baby-box schemes

Of 77 parent-survey respondents almost all had been offered or applied for a baby-box (95%, 73/77), with 89% (65/77) receiving one. Approximately equal proportions of those receiving a box experienced the baby-box schemes in 2017 (29%, 19/65), 2018 (35%, 23/65) and 2019 (35%, 23/65). Lack of nearby collection points, refusal to pay for shipping, lack of availability, and delivery failure explained non-receipt. Most parents had a box shipped directly to their home (77%, 50/65), while others collected it from a community hub (15%, 10/65) or hospital (8%, 5/65). Reasons given for accepting a baby-box involved a) to use as a sleep space (86%, 56/65), b) for the box contents (35%, 23/65), and c) to use for storage (20%, 13/65). Parents had very poor knowledge about the box-provider, most believing this was their NHS Trust (39%, 30/77); only 27% (21/77) understood the scheme was a commercial venture, while 25% (19/77) were unclear who the provider was. Some parents (9%, 7/77) believed the box-provider was a charity.

#### The baby-boxes

Overall parents (88%, 57/65) were satisfied with the construction of the boxes, describing them as ‘sturdy’ and ‘solid’. Some parents who wished to use the box for storage were disappointed there was sometimes no lid or handles (excluded from later versions of box). Most boxes contained a mattress, nappies, baby wipes, a blanket or sheet, and leaflets. Babygro/vest, bib, creams, bath sponge, hat, mittens, socks, breast pads and soft toys were mentioned occasionally. 86% (50/58) of parents that recalled receiving products in their box were either satisfied “I wasn’t expecting any ‘free stuff’”, or somewhat satified; “It seemed a bit sparse in comparison to the Scottish boxes” with the box contents, while 14% (8/58) expressed dissatisfaction: “I don’t believe that what was being advertised was delivered”.

Most parents (68%, 44/65) placed their baby in the box for some part of the day or night, awake or asleep, during 0–3 months of age. Explanations for not using (or ceasing to use) the box included having another place to put the baby, not wanting to put the baby in a box, baby being unsettled in the box, and being unhappy with its construction or location: “Our first night home our daughter went in it and hated it”; “I think it’s not really suitable if you have older children as my son kept tripping over it and fell in it twice (baby wasn’t in it thankfully)...”. Of the 54% (35/65) of parents who regularly (at least once per week) used the box, only 17% (6/35) did so at night-time (baby awake or asleep). The majority of box-use (83%, 29/35) occured during the day-time (baby awake or asleep), e.g. mothers reported using it in the bathroom so they could take a shower. Around 23% (15/65) of box recipients had used the box when visiting friends/family or on holiday, the majority (12/15) doing so regularly: “It is a useful space for baby to sleep when at my mother’s, I leave it at her house. Much safer and better than sleeping in a car seat.”

Following the comments of practitioners, we asked parents whether they had modified the box before using it. Six parents reported tilting the mattress (2/6), or the box itself (3/6), or adding a sleep positioner to the box (1/6).

#### The online education

Parent survey respondents were asked if they were offered educational information, including how to use the box safely: 95% (53/56) received an online video course, 54% (30/56) were given written information, and 7% (4/56) received face-to-face information; 6% (4/65) received no educational information and 8% (5/65) could not recall. Most respondents reported viewing all the videos (85%, 45/53) while 15% (8/53) viewed some or skipped through. Likewise, 73% (22/30) who received written information read everything provided, while 27% did not (8/30). Parents identified the information they had received as covering safe sleeping and SIDS (80%, 45/56); baby-box safety (68%, 38/56); general infant care (43%, 24/56); and antenatal care (30%, 17/56). No parents commented on the quality of the educational materials.

#### Other feedback on the schemes

We asked parents for their opinions on supplying personal details (i.e. website registration) to claim a box. Over half (54%, 35/65) of the participants had concerns about how personal data may be used by the box-provider, a quarter were unconcerned (25%, 16/65) and around a fifth were unsure (22%, 14/65). A few (17%, 11/65) confirmed they were happy to exchange their personal data for a box, however all respondents receiving a box had already completed the online registration. This suggests parents were less aware than practitioners that by signing up for a baby-box they were giving their contact details to a third party and non-EU company.

“I don’t mind my data being used to benefit me but I feel uneasy about companies having sensitive data about me if I don’t know what they are doing with it.”

“I feel like new mothers can be vulnerable, if they are being contacted for sales purposes, and possibly made to feel like they need a certain product for the safety of their baby they may be persuaded to spend money unnecessarily.”

“Hopefully the details wouldn’t be passed on to another company or used inappropriately.”

“I am concerned that other companies would use my data to send me lots of advertising and unnecessary leaflets.”

Parents were asked whether they would recommend the baby-box scheme they experienced to others in their area: 75% (58/77) would, 16% (12/77) would not and 9% (7/77) had no opinion. Nineteen parents offered written comments higlighting evidence gaps around SIDS/SUDI reduction, more pressing areas of need for resource-investment, lack of consistency in schemes and coverage, the need for clearer use instructions, and dissatisfaction with collection points/delivery charges.

“I’m not sure what the point of them is to be honest. There is no evidence they reduce the incidence of SIDS. In this country it appears they are another way to advertise brands to new parents.”

“Felt very frustrated after watching all the videos only then to find out the high price of postage and that the nearest collection point was over 40 miles away!”

“Waste of money. They should put the money used into breastfeeding support. Most people use as a storage box.”

“It’s a fantastic idea - just needs a few improvements!”

## Discussion

Healthcare providers implemented Type-3 baby-box schemes with a commercial partner in England to increase face-to-face contact with parents, and take advantage of the ‘no-cost’ opportunity to provide online educational information. Some practitioners repeated claims that baby-boxes reduce SIDS or enhance safe sleep messaging, and erroneously claimed this was the primary purpose of the scheme in which they were involved. Specific examples were highlighted of how low income families or those in temporary accomodation benefitted from the box and its contents, while the free products were an incentive for families to join the scheme. Practitioners found that box contents were insubstantial or dwindled over time, and while most parents seemed content to receive anything for free, some respondents (both parents and practitioners) considered the boxes to be a waste of money. Practitioners and parents also discovered that ‘free’ baby-boxes came at a cost. Staff invested time in setting up the schemes, producing educational content, informing parents about the scheme, making up and handing out boxes, and explaining how to use them safely. After the initial start-up (when delivery was free) parents invested money and time in obtaining their boxes which some felt wasn’t worth the effort. Practitioners had difficulties storing the boxes and making them available at times when parents could collect them.

Despite practitioners’ concerns about durability, parents found the boxes to be acceptable quality, and most were willing to use them as daytime and occasional sleep spaces for their babies. Practitoners were anxious about parents using the boxes unsafely without face-to-face education, and in some cases these fears were realised when parents placed baby-boxes on unsafe surfaces or put items inside them. A handful of parents acknowledged using boxes in ways that were not recommended for infant sleep. Practitioners were disappointed with the online video education, particularly that parents could gain their box without watching the required videos, and that videos made by the box-provider were not consistent with UK recommendations. Practitioners themselves found the writing of video scripts to be time-consuming and at least one was unhappy with being labelled as an ‘expert’. An issue raised by multiple practitoners was their role in encouraging parents to give personal details to a commercial third party unconnected with their health facility. Given recent incidents with companies unlawfully selling data provided by parents via the UK health-service, [29, 30] some practitioners were wary that new parents may become marketing targets having signed-up for a baby-box. Knowledge of the box-provider was poor among parents with many believing their baby-box was provided by the local health service.

Finally, practitioners and some parents highlighted the changing nature of baby-box schemes since 2016. The way in which parents accessed boxes changed (from free collection to paid shipping) eliminating the incentive for parents to visit community hubs where they could be signposted to other services and offered information, reducing the value of baby-box schemes to many staff. Other practitioners found that while some NHS hospitals disengaged from the scheme, local authority providers (e.g. children’s centres) still promoted them, resulting in conflicting information for parents. Finally the change in the presentation of the baby-box venture, from an educational programme to a rewards scheme troubled practitioners who felt it had now become a baby-club (Type-2 scheme) that was exploiting the partnerships established with the NHS.

### Limitations

There are limitations to this evaluation which must be kept in mind when interpreting the outcomes and recommendations. Only 8/24 NHS Research and Development departments confirmed the evaluation invitation was shared with practitioners in their area, and only 8 individuals volunteered to be interviewed; these 8 interviewees are unlikely to capture the experiences of all NHS staff directly involved in baby-box schemes. Mindful of this limitation we expanded the evaluation to include practitioner surveys which increased the response, but will not have reached all potential respondents. This evaluation therefore reflects the views of a self-selected sample of practitioners and parents that cannot be generalised and potentially presents a biased view [31]. That we obtained responses across a wide range of locations covering all NHS regions where box-schemes were operating, and received a full-spectrum of responses, provides some reassurance that no particular group’s perspective has overwhelmed the findings.

### Recommendations

Based on the above findings Table 2 offers 16 recommendations for healthcare providers considering becoming involved with a baby-box scheme.

**Table 2:** Recommendations for those considering setting-up a baby-box partnership scheme

Those involved in setting-up a baby-box scheme should consider the following recommendations that are based on the issues revealed by this evaluation.	
1	Determine what type of scheme is being proposed (see Fig. 1a-d). Type-2 and Type-4 are entirely commercial.
2	Identify measurable outcomes that are meaningful in the context of the community that will be served by the scheme. Produce a logic model that details the anticipated pathway from box-provision to intended outcomes.
3	Avoid all claims of SIDS-reduction and avoid comparisons with Type-1 (government-run) schemes unless you are implementing a Type-1 scheme.
4	Conduct a robust risk assessment around protection of parents’ data and marketing by third parties, paying particular attention to General Data Protection Regulation requirements.
5	If boxes are provided by a commercial partner, establish a process for regularly monitoring the boxes and free products provided to parents for quality, quantity, and substitutions.
6	Require advance notification of any proposed changes to the box, its contents, any education platform, its presentation to parents, third party partners, data usage etc.
7	If online education is involved in the scheme, consider who writes scripts for this education, where information is sourced from, and how much time is allocated to this. If using secondary video resources, scrutinise their content and confirm they are a good fit for your needs.
8	Decide whether you are happy for parents to receive boxes without viewing the educational videos – if not ensure they must be viewed in full before launching the scheme.
9	Consider whether safety information should be printed on the inside of the box and what written information will be provided with the box. a. Explain to parents why certain safety features are important e.g. lack of lid and handles. b. Explain to parents why certain practices (e.g. replacing the mattress) are unsafe.
10	Determine how boxes will be made available to parents and for what duration, with regular review. If the outcomes of the scheme rely on face-to-face collection of the box, ensure parents will not be offered delivery options for the duration of the scheme.
11	If boxes must be collected face-to-face, consider where boxes will be stored (space required as well as environmental suitability) and who will give them out. Assess how much time should be allocated to do this, bearing in mind that restricting availability will reduce uptake.
12	Establish a mechanism for practitioners and parents to report adverse events (AEs) associated with box-use in a timely manner. Maintain a central repository of AEs with regular review.
13	Regularly review the box provider’s parent-facing website and ensure educational materials are monitored on a regular basis a. Check the range of educational materials online available to parents in your region b. Agree a regular programme of updates to the educational materials you provide c. Identify any changes in presentation of the programme to parents
14	Formalise any agreements with co-partner organisations (e.g. local authorities) and ensure there is clarity about what happens to the scheme should one of you decide to terminate involvement.
15	Secure resources to undertake a rigorous evaluation after an initial pilot phase, benchmarked against the agreed measurable outcomes. Appoint an independent chair for a review committee that oversees the evaluation.
16	Ensure full transparency to parents and staff regarding any commercial provider(s), their interests in establishing the scheme, who will receive parent-data and for what purposes, and the role of the health-provider organisation facilitating the scheme.

## Conclusions

Many assumptions exist about the origins and purpose of baby-boxes; this misinformation needs correcting, especially as it relates to reducing sudden unexpected deaths in infancy and safe infant sleep. Baby-box schemes take multiple forms from those motivated by social welfare to those motivated by commercial profit. The English experience of hybrid (Type-3) partnership schemes between healthcare facilities and commercial box-providers reveals some success stories, along with multiple points of ambiguity, unanticipated difficulty, and concerns for infant safety. It is clear that many of the issues raised by practitioners and parents who responded to this evaluation were not well thought through or agreed with the box-provider before the schemes were launched. Although this evaluation is limited in scope, our findings provide useful information for those considering implementing future programmes involving baby-boxes, wherever they are located.

## Data Availability

Anonymised survey data and redacted interview data are available from the corresponding authors at the Durham University Infancy & Sleep Centre on reasonable request.

## Abbreviations

JISC: Joint Information Systems Committee (not-for-profit provider of digital resources for UK Higher Education institutions)
NHS: National Health Service (UK)
SIDS: Sudden Infant Death Syndrome (unexplained infant death)
SUDI: Sudden Unexpected Death in Infancy (explained and unexplained infant deaths)
UK: United Kingdom
US: United States
